# The prevalence and psychosocial risk factors of chronic low back pain in KwaZulu-Natal

**DOI:** 10.4102/phcfm.v14i1.3134

**Published:** 2022-01-25

**Authors:** Morris Kahere, Themba Ginindza

**Affiliations:** 1 Discipline of Public Health Medicine, School of Nursing and Public Health, Faculty of Health Sciences, University of KwaZulu-Natal, Durban, South Africa

**Keywords:** chronic low back pain, risk factors, psychological predictors, prevalence, KwaZulu-Natal

## Abstract

**Background:**

Chronic low back pain (CLBP) is the leading cause of disability and has been extensively investigated in high-income countries (HICs), with little done in low-and middle-income countries. Biomechanical stressors do not have a major pathogenic role, but psychosocial predisposition is important. The occurrence and progression of CLBP are significantly affected by psychosocial risk factors. Guidelines recommend the early identification of psychosocial factors that could predict CLBP.

**Aim:**

To determine the prevalence and psychosocial risk factors for CLBP amongst adults in KwaZulu-Natal, South Africa.

**Setting:**

The study was conducted at five randomly selected public hospitals in KwaZulu-Natal.

**Methods:**

Analytical cross-sectional hospital-based study utilising a self-administered questionnaire to collect data on (1) sociodemographic, (2) disability, (3) fear-avoidance beliefs and (4) illness behaviour. The Statistical Package for the Social Sciences (SPSS) 24.0 was used for data cleaning and descriptive statistics. Chi-square test was used for categorical variables. Standard Edition of the Statistical Software for Data Science version 17.0 (STATA 17.0 SE) was used to identify risk factors using the logistic regression analysis. A *p*-value of ≤ 0.05 was deemed statistically significant.

**Results:**

Overall prevalence of CLBP was 22.2% (95% confidence interval [CI]: 18.8–25.9). Females had a higher prevalence of CLBP than males, 23.9% (95% CI: 19.4–28.9) and 19.7% (95% CI: 14.8–25.5), respectively; however, the difference was not significant *p* = 0.243. The multivariate regression analysis identified the following risk factors: female gender, middle-aged adults 38–47 years, obesity, disease conviction, affective disturbance, denial and fear-avoidance behaviour-work subscale.

**Conclusion:**

There is a high prevalence of CLBP amongst the study participants. Psychosocial factors (disease conviction, affective disturbance and fear-avoidance behaviour about work) are significant predictors of CLBP.

## Background

Low back pain (LBP) is a leading cause of disability and the most prevalent musculoskeletal condition globally.^1^ Although, worldwide, the prevalence of LBP is high, with up to 84% of people reporting at least one episode in their lifetime,^2^ most people recover within a few weeks.^3,4^ However, about 10% – 40% of all LBP patients will have recurrent episodes and develop chronic low back pain (CLBP).^5^ The majority of disability and financial burden associated with LBP is attributed to this minority of the population who develop CLBP.^6^ Globally, the prevalence of CLBP is estimated at 19.1%^7^ and is expected to increase in low- and middle-income countries (LMICs), where healthcare systems are mostly focussed on communicable disease control and insufficiently equipped to deal with the increasing burden of non-communicable diseases.^8,9^ In Africa, the prevalence of LBP is 47%.^10^

Defined as ‘pain, muscle tension, or stiffness localised below the costal margin and above the inferior gluteal folds, with or without leg pain (sciatica)’, LBP is described as chronic when it persists for 12 weeks or more.^11^ Most LBP cases are non-specific, which means there is no identifiable pathoanatomic cause.^12^ Non-specific LBP diagnosis is based on the exclusion of a specific pathology and is found in about 90% of all LBP patients.^13^ Non-specific LBP is therefore difficult to manage and often easily progresses to CLBP. Notwithstanding the projected increase in CLBP burden, there is little evidence on CLBP in sub-Saharan Africa (SSA). The majority of studies in SSA, mostly conducted in Nigeria and South Africa, have focused on LBP.

As reported by the Cochrane Back Review Group,^14^ identification of factors that influence outcome of acute and/or subacute LBP cases is a major challenge to improving prognosis. In addition to the well-understood biomedical and biomechanical models of LBP, certain psychosocial factors have been significantly associated with poor outcomes.^15^ Recently published guidelines for LBP emphasise the early identification of psychosocial factors that could predict chronicity of acute LBP cases.^16,17,18,19^ These psychosocial factors include fear avoidance,^20^ abnormal illness behaviour,^21^ catastrophising,^22^ low self-efficacy^16^ and depression.^23^ In addition to psychological distress, pain-related fear arbitrates the relationship between pain and disability.^24^

Although several models have attempted to explain why disabling back pain develops and why it persists, the fear-avoidance belief model (FABM)^25^ has gained widespread recognition and validation across physical therapy literature.^26,27^ The FABM describes how the experience of LBP symptoms can result in negative cognitive, emotional, and behavioural responses.^27^ Avoidance is a maladaptive response that can cause a patient to avoid certain activities that are anticipated to exacerbate the perceived symptoms.^27^ This ultimately leads to diminished physical and social activities, aggravation of the fear and avoidance behaviour persistence of disability and adverse physical and psychological consequences.^28^ A vicious cycle proceeds, in which the fear-avoidance behaviour leads to depression and persistent physical disability that sequentially worsens the perception of pain.^27^

The perception or experience of LBP ignites a cognitive representation that is based on the individual’s beliefs about LBP. Low back pain-related fear is a reactive common-sense problem-solving response that is often proceeded by a self-regulatory process where the clinical outcome of the coping response is appraised, and the sequel of the appraised coping strategy feeds back into the LBP representation to guide future coping strategies.^27,29,30^ Emotion-directed coping manages the cognitive emotions associated with the LBP experience. Low back pain perceived as having adverse effects elicits a fear response that alters behaviour.^29^ Illness behaviour is hypothesised to influence clinical outcome within the common-sense model and the self-regulatory models^30^ and has been shown to predict outcome in LBP.^21^

The clinical practice guideline published in South Africa^31^ in 2020 is based on studies conducted in high-income countries (HICs) and focuses only on acute and subacute LBP. A recent study by Major-Hesloot et al.,^32^ reported that the current management of LBP in primary care appears to be ineffective and does not conform to the guidelines. South Africa’s National Development Plan and health policy seek to improve health outcomes.^33^ However, not much is known on the prevalence/risk factors of CLBP, a leading driver of disability. It is in this backdrop that we conducted this cross-sectional study in KwaZulu-Natal (KZN), and the purpose of this study was to determine the association between chronic LBP and gender, age, body mass index, level of education, annual household income, abnormal illness behaviour and fear-avoidance beliefs.

## Methods and materials

### Study design and setting

A hospital-based analytical cross-sectional study – was conducted in five provincial hospitals (Prince Mshiyeni Memorial Hospital, Mahatma Gandhi Memorial Hospital, Clairwood Hospital, Hillcrest Hospital and Addington Hospital) in eThekwini municipality of KZN, South Africa. The public sector provides healthcare services to the majority of the South African population through a referral district health system where provincial hospitals provide tertiary care. The eThekwini municipality, which includes the city of Durban, is the only metropolitan and most populous of the 11 municipalities in KZN with a population of 3 158 000 and has 17 provincial hospitals.

### Participants

Participants aged 18 years and above (adult males or females) were enrolled to the study. All participants included in the study were attending outpatient care at the selected study sites for health services, willing to sign the informed consent, and who self-reported current experience or current history of LBP. Participants were excluded if they were below the age of 18 years, have a known specific cause of LBP such as tuberculosis (TB) or cancer, mentally ill patients and/or those who refused to sign the informed consent. Participants were recruited using a systematic random sampling technique where every third person (after the first participant was selected using the lottery method) presenting at the selected study site was selected until the required calculated sample size was achieved.

### Sample size

To estimate the prevalence of the outcomes of interest (CLBP), assuming 95% confidence and an acceptable margin of error of 5% and maximum variability, that is, 50% (given unknown prevalence), a sample size of 384 subjects was required. The sample was further increased by a margin of 10% to account for potential non-response and multiplied by a design effect (D) of 1.3. The final sample size of the entire study was 650 participants. A detailed account of the methodology of the study is found in the published protocol.^34^ However, based on the inclusion criteria of this portion of the study, only 554 participants satisfied the criteria and were selected for further analysis.

### Study instruments

#### Fear-avoidance belief

We used a self-administered questionnaire with four sections to gather participants sociodemographic characteristics, fear avoidance, disability and illness behaviour. A fear-avoidance belief questionnaire (FABQ) developed by Waddell et al.^35^ was used to assess the risky effects of fear-avoidance behaviour on CLBP disability. The FABQ is a 16-item Likert-scale questionnaire with item scores ranging between 0 (strongly disagree) and 6 (strongly agree) representing the extent of agreement to given statements. Higher scores indicate increased levels of fear-avoidance beliefs. The FABQ demonstrated high test–retest reliability and internal consistency.^35^ The principal component analysis of the questionnaire identified two important factors, namely fear-avoidance beliefs about physical activity and fear-avoidance beliefs about work with internal consistence of 0.77 and 0.88, respectively, and accounting for a respective 16.5% and 43.7% of the total variance.^35^ This is the established validity in the population for which it was designed and not in a South African population.

#### Illness behaviour

Illness behaviour was measured using the Pilowsky and Spence 62-item Illness Behaviour Questionnaire (IBQ).^36^ The self-administered IBQ comprised the following seven subscales: (1) general hypochondriasis – high score indicating a phobic anxious concern about health, (2) disease conviction – high scores indicate preoccupation with symptoms and a strong affirmation that the disease is present, (3) psychologic versus somatic perception of illness – high score indicates that the participant perceives pain in psychological terms, whereas a lower score suggests a tendency to somatise, (4) affective inhibition – high score indicates that the participant is unable to express personal or negative feeling to others, (5) affective disturbance – high scores indicate excessive anxiety and depression, (6) denial – high score indicates that the participants tend to deny life stresses or attributes them to physical problems and (7) irritability – high score indicates that the participant is bothered by the feeling of anger, particularly in interpersonal situations.^36,37^ The subscales consist of at least five binary response items. A detailed presentation of IBQ’s validity and reliability is provided in the Manual for the Illness Behaviour Questionnaire.^38^

#### Statistical analysis

The IBM Statistical Package for Social Sciences (SPSS) software version 27.0 for Windows (IBM Corp., Armonk, NY, United States [US]) was used for data cleaning and descriptive statistics. Data were analysed using Stata 13.0SE (Stata corp. College Station, Texas, US). Participants sociodemographic information were analysed using descriptive statistics, presented as frequencies (*n*), proportions (%) and the associated 95% confidence interval (CI). A chi-square test of independence was performed to determine whether there was a statistically significant relationship between categorical variables. Odds ratios (unadjusted and adjusted) and 95% CIs for potential psychosocial risk factors of CLBP were estimated using a logistic regression model. All variables in the univariate analysis were included into the multivariable model. The level of significance was set at *p* ≤ 0.05 in the multivariate model.

## Results

### Sociodemographic characteristics of the study population

A total of 554 participants with a current or previous history of LBP were enrolled into the study (Table 1). The ratio of males to females was approximately 2:3 (41.2%:58.8%). The mean (± standard deviation [s.d.]) age for enrolled participants was 45.8 (±10.7) years, where majority (51.8%) of them were middle-aged adults aged between 28–37 years and 38–47 years. There were 202 (36.5%) overweight participants, with only a third (166, 30.0%), having normal body mass index (BMI). Obesity was observed in 135 (24.4%) participants. The highest level of education was normally distributed amongst the study participants. The poor and the low emerging income category constituted the majority of the study population, 104 (18.8%) and 273 (49.3%), respectively. Only 106 (19%) participants reported LBP associated with leg pain (sciatica). The onset of LBP symptoms was gradual without injury in 347 (62.6%) participants, with only 104 (18.8%) reporting severe symptoms. Only about a fifth of the participants, 115 (20.8%) reported constant pain, with half (50.5%) reporting interference of LBP symptoms with activities of daily living.

**TABLE 1:** Sociodemographic characteristics of the study population (*n* = 554).

Demographics	*n*	%	Mean ± s.d.
**Gender**			
Male	228	41.2	-
Female	326	58.8	-
**Age**			45.8 ± 10.7
18–27	19	3.4	-
28–37	111	20.0	-
38–47	176	31.8	-
48–57	163	29.4	-
58+	85	15.3	-
**Body mass index**			
Under weight	51	9.2	-
Normal	166	30.0	-
Overweight	202	36.5	-
Obese	135	24.4	-
**Level of education**			
No formal education	106	19.1	-
Primary	194	35.0	-
Secondary	144	26.0	-
Tertiary	110	19.9	-
**Annual household income**			
Poor	104	18.8	-
Low emerging middle	273	49.3	-
Emerging middle	96	17.3	-
Realised middle	49	8.8	-
Upper middle class	32	5.8	-
**Sciatica**			
Yes	106	19.1	-
No	448	80.9	-
**LBP onset**			
Gradually without injury	347	62.6	-
Abruptly without injury	120	21.7	-
Gradually after an injury	52	9.4	-
Abruptly after an injury	17	3.1	-
**LBP severity**			
Mild	303	54.7	-
Moderate	147	26.5	-
Severe	104	18.8	-
**LBP frequency**			
Seldom	70	12.6	-
Occasionally	300	54.1	-
Frequently	69	12.5	-
Constantly	115	20.8	
**LBP progression**			
Still the same	87	15.7	-
Getting better	29	5.2	-
Getting worse	93	16.8	-
Comes and goes	345	62.3	-
**Interference with ADL**			
Yes	280	50.5	-
No	274	49.5	-

s.d., standard deviation; LBP, low back pain; ADL, activities of daily living.

### Prevalence of chronic low back pain

The overall prevalence of CLBP amongst adult males and females aged 18 years and above with a previous or current history of LBP was 22.2% (95% CI: 18.8–25.9; Table 2). Overall, the prevalence of CLBP was higher amongst females to males, 23.9% (95% CI: 19.4–28.9) and 19.7% (95% CI: 14.8–25.5), respectively, although the difference was not statistically significant, *p* = 0.243. However, amongst older adults aged 58 years and above, males had a higher CLBP prevalence than females, 33.3% (95% CI: 16.5–54.0) and 31.0% (95% CI: 19.5–44.5), respectively. The chi-square test of independence showed that gender (*χ*^2^ = 1.4, *df* = 1, *p* = 0.243), age (*χ*^2^ = 6.9, *df* = 4, *p* = 0.143) and sciatica (*χ*^2^ = 1.6, *df* = 1, *p* = 0.690) were not significantly associated with CLBP. All the other variables were significantly associated with CLBP (Table 2).

**TABLE 2:** Prevalence and characteristics of chronic low back pain (*n* = 554).

Variable	Total (*n*)	Chronic Low Back Pain (CLBP)			*χ* ^2^	*p*
		*n*	Prevalence (%)	95% CI		
**Gender**						
Male	228	45	19.7	14.8–25.5	**1.363**	**0.243**
Female	326	78	23.9	19.4–28.9		
**Age**						
18–27	19	6	31.6	12.6–56.6	**6.877**	**0.143**
28–37	111	21	18.9	12.1–27.5		
38–47	176	35	19.9	14.3–26.6		
48–57	163	34	20.9	14.9–27.9		
58+	85	27	31.8	22.1–42.8		
**Body mass index**						
Under weight	51	7	13.7	5.7–26.3	**38.303**	**< 0.001**
Normal	166	12	7.2	3.8–12.3		
Overweight	202	62	30.7	24.4–37.6		
Obese	135	42	31.1	23.4–39.6		
**Level of education**						
No formal education	106	32	30.2	21.7–39.9	**21.952**	**< 0.001**
Primary	194	30	15.5	10.7–21.3		
Secondary	144	23	16.0	10.4–23.0		
Tertiary	110	38	34.6	25.7–44.2		
**Annual household income**						
Poor	104	36	34.6	25.6–44.6	**14.404**	**0.006**
Low emerging middle	273	49	18.0	13.6–23.0		
Emerging middle	96	18	18.8	11.5–28.0		
Realised middle	49	10	20.4	10.2–34.3		
Upper middle class	32	10	31.3	16.1–50.0		
**Sciatica**						
Yes	106	22	20.8	13.5–29.7	**0.159**	**0.690**
No	448	101	22.54	18.8–26.7		
**LBP onset**						
Gradually without injury	347	100	28.8	24.1–33.9	**25.561**	**< 0.001**
Abruptly without injury	120	8	6.7	2.9–12.7		
Gradually after an injury	52	10	19.2	9.6–32.5		
Abruptly after an injury	17	5	29.41	10.3–56.0		
**LBP severity**						
Mild	303	6	2.0	0.7–4.3	**173.234**	**< 0.001**
Moderate	147	56	38.1	30.2–46.5		
Severe	104	61	58.7	48.6–68.2		
**LBP frequency**						
Seldom	70	0	-	0.0–0.0	**439.755**	**< 0.001**
Occasionally	300	0	-	0.0–0.0		
Frequently	69	16	23.2	13.9–34.9		
Constantly	115	107	93.0	86.8–97.0		
**LBP progression**						
Still the same	87	10	11.5	5.7–20.1	**351.847**	**< 0.001**
Getting better	29	16	55.2	35.7–73.6		
Getting worse	93	85	91.4	83.8–96.2		
Comes and goes	345	12	3.5	1.5–5.4		
**Interference with ADL**						
Yes	280	111	39.6	33.9–45.6	**99.697**	**< 0.001**
No	274	12	4.4	2.3–7.5		

CI, confidence interval; LBP, low back pain; ADL, activities of daily living.

### Psychosocial risk factors associated with chronic low back pain

Table 3 shows psychosocial risk factors associated with CLBP. Based on the univariate analysis, overweight (odd ratios [OR]: 2.8, 95% CI: 1.2–6.5, *p* = 0.018) and obesity (OR: 2.8, 95% CI: 1.2–6.8, *p* = 0.020) were the only risk factors that were positively associated with CLBP. Primary (OR: 0.4, 95% CI: 0.2–0.6, *p* < 0.001) and secondary (OR: 0.4, 95% CI: 0.2–0.7, *p* = 0.001) level of education were inversely associated with CLBP. General hypochondriasis, disease conviction, psychosocial versus somatic focusing, affective inhibition, affective disturbance, irritability and denial were positively associated with CLBP. After multivariable adjustment, the female gender (adjusted odds ratio [aOR]: 12.4, 95% CI: 3.1–49.8, *p* < 0.001), the age category 38–47 years (aOR: 23.4, 95% CI: 1.2–453.4, *p* = 0.037), obesity (aOR: 8.7, 95% CI: 1.0–74.1, *p* = 0.048), disease conviction (aOR: 19.1, 95% CI: 6.7–55.0, *p* < 0.001), affective disturbance (aOR: 3.3, 95% CI: 1.2–8.8, *p* = 0.020), denial (aOR: 4.2, 95% CI: 1.6–11.1, *p* = 0.004) and fear-avoidance belief work sub-scale (aOR: 1.2, 95% CI: 1.0–1.3, *p* = 0.029) remain positively significantly associated with CLBP. Unexpectedly, no formal education was inversely associated with CLBP (aOR: 0.2, 95% CI: 0.1–1.0, *p* = 0.049).

**TABLE 3:** Association of chronic low back pain and risk factors (*n* = 554).

Variable	Univariate			Multivariate		
	OR	95%CI	*p*	aOR	95%CI	*p*
**Gender**						
Male	1	ref	-	1	ref	-
Female	1.3	0.9–1.9	0.244	12.4	3.1–49.8	< 0.001*
**Age**						
18–27	1	ref	-	1	ref	-
28–37	0.5	0.2–1.5	0.215	7.0	0.4–128.4	0.191
38–47	0.5	0.9–1.5	0.241	23.4	1.2–453.4	0.037*
48–57	0.6	0.2–1.6	0.290	11.7	0.7–204.2	0.091
58+	1.0	0.4–2.9	0.987	4.4	0.3–70.0	0.296
**Body mass index**						
Under weight	1	ref	-	1	ref	**-**
Normal	0.5	0.2–1.3	0.158	0.4	0.1–3.2	0.398
Overweight	2.8	1.2–6.5	0.018	3.4	0.5–21.7	0.204
Obese	2.8	1.2–6.8	0.020	8.7	1.0–74.1	0.048*
**Level of education**						
No formal education	0.8	0.5–1.5	0.494	0.2	0.1–1.0	0.049
Primary	0.4	0.2–0.6	0.000	0.4	0.1–1.6	0.189
Secondary	0.4	0.2–0.7	0.001	0.5	0.1–2.3	0.378
Tertiary	1	ref	-	1	ref	-
**Annual household income**						
Poor	1.2	0.5–2.7	0.725	1.0	0.1–8.6	0.983
Low emerging middle	0.5	0.2–1.1	0.076	0.4	0.1–2.6	0.317
Emerging middle	0.5	0.2–1.3	0.143	0.35	0.1–3.4	0.396
Realised middle	0.6	0.2–1.6	0.272	2.5	0.2–34.8	0.501
Upper middle class	1	ref	-	1	ref	-
**Illness behaviour**						
General hypochondriasis	4.0	3.2–5.0	0.000	1.2	0.6–2.2	0.656
Disease conviction	17.2	10.3–28.7	0.000	19.1	6.7–55.0	< 0.001*
Psychosocial vs somatic	9.6	6.5–14.3	0.000	0.6	0.2–1.8	0.388
Affective inhibition	9.7	6.5–14.5	0.000	1.3	0.5–3.6	0.615
Affective disturbance	15.0	9.1–24.6	0.000	3.3	1.2–8.8	0.020*
Irritability	9.5	6.4–14.2	0.000	1.4	0.5–3.5	0.510
Denial	1.0	6.6–15.0	0.000	4.2	1.6–11.1	0.004*
**Fear avoidance belief**						
FAB – physical activity	1.0	0.9–1.0	0.766	1.1	0.9–1.4	0.248
FAB – work	1.0	0.9–1.0	0.524	1.2	1.0–1.3	0.029*

CI, confidence interval; OR, odds ratio; aOR, adjusted odds ratio.

* , Statistically significate *p* < 0.05.

## Discussion

The aim of this cross-sectional study was to investigate the prevalence of CLBP and its associated psychosocial risk factors for chronicity amongst adult LBP participants. In this study, the prevalence of CLBP is high (22.2%). Stratified by gender, females had a higher prevalence (23.9%) of CLBP than males (19.7%); however the difference was not statistically significant, *p* = 0.243. The highest prevalence of CLBP was observed amongst those that reported having constant pain, (93.0%) followed by those who reported progressive worsening of symptoms (91.4%). The prevalence of CLBP amongst those with severe symptoms was 58.7%, and that which interfered with activities of daily living was 39.6%. The multivariate binary logistic regression analyses showed that the main predictors of CLBP amongst adults with a positive history of LBP were female gender, middle-aged adults aged 38–47 years, obesity, disease conviction, affective disturbance, denial and fear-avoidance behaviour about work.

The observed higher CLBP prevalence amongst females compared to males in this study concurs with several previous studies.^7,39,40,41,42,43^ The current study shows that the prevalence of CLBP increased with increasing age, which is in line with other similar cross-sectional studies.^42,43,44^ This observation can be attributed to increased psychological distress associated with increasing age.^45^ According to a recent study by Sendra et al.,^46^ social media technology has provided a platform for health communication, and those with access to these sources of social learning are exposed to more misinformation about the fragility of the spine that increases their fear beliefs. Sendra et al. also found that females are more sociable than their male counterparts and hence are at risk for more exposure to this misinformation.^46^ This concurs with the findings of our study that found increased prevalence amongst the female gender.

The multivariate analysis of this study shows that the age category 38–47 years was the strongest predictor of CLBP (aOR = 23.4). Considering the mean age of 45 years in our study, the demographic dynamics the advent of technology and the fourth industrial revolution in the African context, this finding is suggestive to be a result of a myriad of factors. These include: employment status, job satisfaction levels, compensation issues, lack of social support that can result in depression, anxiety, psychological distress affecting coping strategies and abnormal illness behaviours.^15^ This concurs with La Touche et al.^47^ study that also reported that CLBP was associated with the less than 45-year-old adults. Poor postural ergonomics amongst this age category^48^ can also play a role in the development of CLBP. Early identification of these factors at the primary care level is important because early and specific interventions can be developed to reduce the chances of chronicity.

Disease conviction was the second strongest risk factor amongst all and the strongest amongst the psychological risk factors of CLBP (aOR = 19.1). This is indicative of poor health literacy in which pain is interpreted as a sign of a major health problem. Disease conviction can result in increased distress and affect pain by reducing the patient’s ability to adapt to the pain.^49^ This is in line with previous studies by Dworkin et al.,^50^ Keefe et al.,^37^ Feuerstein et al.^51^ and Waddell et al.^52^ who reported that high scores of disease conviction were significant predictors of CLBP. In order to prevent the impact of disease conviction, improvements in health literacy should be a priority, and so also health education and promotion especially in underserved communities, as stipulated in the sustainable development goals for 2030.^53^ Again, early identification of the sources of this belief system can help in designing specific intervention measures.

In line with the findings of our study, Rush et al. reported a positive association of back pain with anxiety and depression.^54^ According to Jazaieri et al., affective disturbance is the disruption in the multisystem response (subjective experience, expressive behaviour, physiology) of emotions, moods and stress responses.^55^ However, because of the cross-sectional design of our study, it is unclear whether anxiety and depression causes LBP or aggravates it or if LBP influences anxiety and depression. Future studies on this subject should consider prospective study designs to be able to draw inferences of causality. According to the results of the present study, denial was a significant predictor of CLBP. This concurs with Keefe et al. who also reported similar findings.^37^ Similarly, Waddell et al.^52^ in their prospective study, reported that denial emerged as independent predictors of CLBP. Denial can interfere with interventions and coping strategies as the patient accepts or rejects the aching lower back.^56^

According to this study, fear-avoidance behaviour about work was a significant predictor of CLBP (aOR = 1.2). This concurs with the findings of other previous studies.^57,58^ This observation can be explained by the vicious cycle.^27^ Fear-avoidance beliefs can result from a number of factors, such as sociocultural beliefs, beliefs of healthcare professionals, community health literacy, awareness, education and promotion.^59^ The current guidelines in South Africa recommend early identification of these fear beliefs at primary healthcare level through routine yellow flag assessment. Measures should be put in place to monitor adherence to guidelines by the healthcare service providers, as Major-Hesloot et al. have reported that management of LBP at primary health care level do not conform to guidelines.^32^

## Strengths and limitations

To the best of our knowledge, this is the first study conducted in the sub-Saharan region investigating the psychological risk factors of CLBP. The results of this study provide strategic local data or information and could serve as an important tool for informing policy. The limitations of this study include the use of the illness behaviour questionnaire that has been criticised because of the relative paucity of validity and reliability in capturing the desired outcome and lacks psychometric satisfaction. We cannot rule out recall bias because of the use of self-report data in this study. The results of this study cannot be generalised to other populations of pain patients. It would have been better if the study had a control group to compare the differences in the psychological properties between the symptomatic and asymptomatic population groups. This study focused only on the pain-related psychological aspects. Future studies should consider looking at other stresses including job satisfaction, financial stress, social or family-related stresses. The study was also limited because of the use of tools that were all in English requiring interviewers translating them. Non-standardised translation can potentially introduce bias. In addition, we cannot rule out recruitment bias, as all the study participants had LBP, and thus the high prevalence of CLBP is not a surprise. Nevertheless, this does not take away from the value of the psychosocial risk factors.

## Conclusion

This study concluded that the prevalence of CLBP is high and is comparable to the prevalence in other HICs. Abnormal illness behaviour and fear-avoidance beliefs were identified as important predictors of CLBP. This study provides important evidence to guide future prevention and management strategies to alleviate the burden of CLBP in South Africa. Risk assessment is key to planning prevention programmes. In addition to red flag assessment, the clinical guidelines for LBP should emphasise yellow flag screening using validated risk stratification tools. Against this backdrop, legislation and enforcement to guideline adherence by primary healthcare workers should be implemented. Cognitive behavioural therapy professionals should be made available and accessible to all. In case of shortage of skills, training of more qualified personnel is recommended to close this gap. Management guidelines should emphasise a multidisciplinary intervention approach to address all the possible risk factors (biomedical, biomechanical and biopsychosocial) associated with CLBP. Information provision and education based on the biopsychosocial model are effective strategies in modifying beliefs about CLBP and reducing its associated socio-economic burden. Funders, policy or decision makers, community stakeholders and other involved actors should ensure sufficient resource allocation to deal with increasing burden of non-communicable diseases.
